# Luteinizing Hormone Levels Relate to the Unfavorable Pathology of Prostate Cancer

**DOI:** 10.3390/jcm9051281

**Published:** 2020-04-29

**Authors:** Se Young Choi, Byung Hoon Chi, Wonchul Lee, Bumjin Lim, Dalsan You, Choung-Soo Kim

**Affiliations:** 1 Department of Urology, Chung-Ang University Hospital, Chung-Ang University College of Medicine, 102 Heukseok-ro, Dongjak-gu, Seoul 06973, Korea; 2 Department of Urology, Asan Medical Center, University of Ulsan College of Medicine, 88 Olympic-ro 43-gil, Songpa-gu, Seoul 05505, Korea

**Keywords:** testosterone, sex hormone-binding globulin, luteinizing hormone, follicle-stimulating hormone, prostate neoplasms

## Abstract

Purpose: This study analyzed the association between sex hormone concentrations and stage/condition in patients with prostate cancer. Materials and methods: The concentrations of sex hormones, including testosterone (total, free, and bioavailable), sex hormone-binding globulin (SHBG), luteinizing hormone (LH), and follicle-stimulating hormone (FSH), were measured in 415 patients diagnosed with prostate cancer. Differences in serum hormone concentrations after receiving androgen deprivation therapy (ADT) and after withdrawal from ADT were evaluated. Pathologic characteristics were assessed in the 225 patients unexposed to ADT with a history of radical prostatectomy. Logistic regression analysis was performed to identify factors predictive of unfavorable pathology (Grade ≥3, ≥T3a, or N1). Results: Of the 415 prostate cancer patients, 130 (31.3%) were assessed before treatment, 171 (41.2%) after surgery, 35 (8.4%) after biochemical recurrence, and 59 (14.2%) during ADT, whereas 20 (4.8%) had castration-resistant prostate cancer. FSH was significantly lower after compared to before prostatectomy (3.229 ± 4.486 vs. 5.941 ± 7.044 mIU/mL, *p* < 0.001). LH, FSH, and testosterone decreased significantly 3 months after starting ADT, but increased 3 months after ADT withdrawal, whereas SHBG was unchanged. Multivariate analysis showed that high LH (odds ratio [OR]: 1.59, 95% confidence interval [CI]: 1.03–2.47, *p* = 0.0376) and prostate-specific antigen (PSA) (OR: 1.13, 95% CI: 1.03–1.24, *p* = 0.0133) concentrations were significantly associated with a high risk of unfavorable pathology. Conclusions: Sex hormones, including LH, FSH, and testosterone, were affected by ADT. The FSH level decreased after radical prostatectomy. High baseline LH concentration in patients unexposed to ADT was associated with an unfavorable pathology.

## 1. Introduction

The effect of castration, which eliminates androgen, has been proved to regulate prostate cancer development levels [1]. Luteinizing hormone releasing hormone (LHRH) agonists are frequently used to treat patients with metastatic disease or recurrence, and LHRH antagonists are used as androgen deprivation agents [2]. LHRH antagonists directly inhibit LHRH receptors in the pituitary gland and rapidly reduce the production of testosterone, luteinizing hormone (LH), and follicle-stimulating hormone (FSH) [3]. By comparison, LHRH agonists initially stimulate the production of LH, increasing testosterone production for 2 weeks [3]. Three days after LHRH agonist administration, LH and FSH concentrations peak, with these hormones being about 10- and 5-fold higher, respectively, than their baseline levels [4]. After the peak, the hypothalamus-pituitary-gonadal axis participates in negative-feedback through the down-regulation of LHRH receptor and the suppression of gonadotropin production [5]. Androgens initially regulate androgen receptor and androgen receptor activity and then, androgen receptor mutation/amplification finally leads to castration-resistant prostate cancer (CRPC) development. The aim of androgen deprivation therapy (ADT) is not to suppress androgen by itself, but to suppress androgen receptor signaling. There are lots of studies about androgen receptor variants, but relatively less is known about the associations of sex hormones with the growth, proliferation, or progression of prostate cancer.

Prostate-specific antigen (PSA) is commonly used to screen patients for prostate cancer during active surveillance and to assess biochemical recurrence (BCR). However, PSA has low specificity in the screening of some patients and in monitoring progression during active surveillance. Understanding the roles of sex hormones in prostate cancer may compensate for these limitations. For example, low testosterone levels may be related to a high risk of prostate cancer [6], and a saturation model suggested that testosterone replacement may be safe in prostate cancer patients [7]. Dysregulation of FSH may be associated with the development and progression of prostate cancer [8]. A study of 1170 men with prostate cancer found that impaired LH signaling may be related to a lower cancer risk but a higher cancer-specific mortality rate [9]. As a major carrier of testosterone, sex hormone-binding globulin (SHBG) can influence testosterone uptake and action. This study assessed the relationship between the concentration of each sex hormone and the condition of prostate cancer. 

## 2. Materials and Methods 

**Figure 1:**
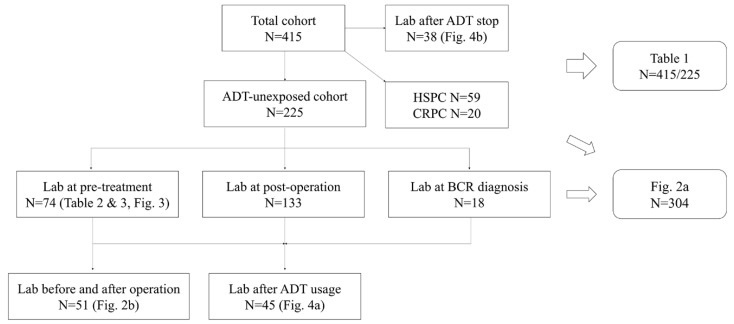
Study flow diagram. ADT, androgen deprivation therapy; BCR, biochemical recurrence; CRPC, castration-resistant prostate cancer; HSPC, hormone-sensitive prostate cancer.

This study protocol was approved by the ethical review board at our institution. Examination of medical records retrospectively identified 415 patients who had been diagnosed with prostate cancer and underwent measurements of serum sex hormone concentrations at the same time between 2016 and 2018. Sex hormones measured included total testosterone, SHBG, LH, and FSH. Free and bioavailable testosterone levels were calculated using testosterone, albumin, and SHBG levels [10]. The condition of each prostate cancer patient was recorded, including clinical TNM (Tumor, Node, Metastasis) stage, factors obtained at biopsy, and medication use at the time of sex hormone measurements. Differences in serum hormone levels after receiving androgen deprivation therapy (ADT) and after withdrawal from ADT were evaluated. In the total cohort, 225 patients were unexposed to ADT and the remaining 73 patients were exposed to ADT at the timepoint of sample collection. In the total cohort, there were 13 patients who underwent radiotherapy or radiotherapy with ADT. At the timepoint of sample collection, there were 59 patients with hormone-sensitive prostate cancer (HSPC) and 20 patients with castration-resistant prostate cancer (CRPC). Pathologic characteristics were evaluated in the subgroup of 225 patients who underwent radical prostatectomy but were unexposed to ADT. This subgroup included 74 patients assessed prior to treatment, 113 evaluated after surgery, and 18 diagnosed with BCR. Figure 1 shows the flow diagram of this study.

Blood samples were obtained before 11:00 am. SHBG (Immunotech, Beckman Coulter, Prague, Czech Republic), LH (DIAsource, DIAsource Immunoassays S.A, Ottignies-Louvain-la-Neuve, Belgium), FSH (DIAsource, DIAsource Immunoassay), and testosterone (Cisbio, Cisbio Bioassays, Codolet, France) concentrations were assayed using commercially available kits. 

The baseline characteristics of the patients and tumors were reported as means ± standard deviations with interquartile ranges or as frequencies with percentages. Normal distribution of data was determined using the Kolmogorov-Smirnov test and pairs of groups were compared using T-tests or the Mann Whitney U-test. Wilcoxon signed rank tests were used to compare hormone concentrations before and after surgery or change in ADT. Factors predictive of unfavorable pathology, including Grade ≥3, pathologic stage ≥T3a, or pathologic stage N1, were assessed in untreated patients by logistic regression analysis. Factors significant on univariate analyses (*p* < 0.05) were entered into multivariate analysis to determine factors that were independently predictive of unfavorable pathology. All statistical analyses were performed using IBM SPSS Statistics Version 21 (IBM Corporation, Somers, NY, USA). All *p* values were two-tailed, with *p* < 0.05 considered statistically significant.

## 3. Results

The baseline characteristics of the total cohort (n = 415) and ADT-unexposed cohort (n = 225) at the first laboratory examination are presented in Table 1. Of the patients in the total cohort, 14.2% had HSPC using ADT and 4.8% had CRPC at the first laboratory examination. Mean initial PSA was 73.5 ± 287.7 ng/mL in the total cohort and 11.8 ± 16.7 ng/mL in the ADT-unexposed cohort. Of these cohorts, 54.0% and 44.0% of patients, respectively, had clinical grade ≥3. The percentages of patients with T ≥3 (42.7% vs. 29.2%), N (16.7% vs. 0.4%), and M (15.5% vs. 0%) stage were higher in the total than in the ADT-unexposed cohort. 

**Figure 2:**
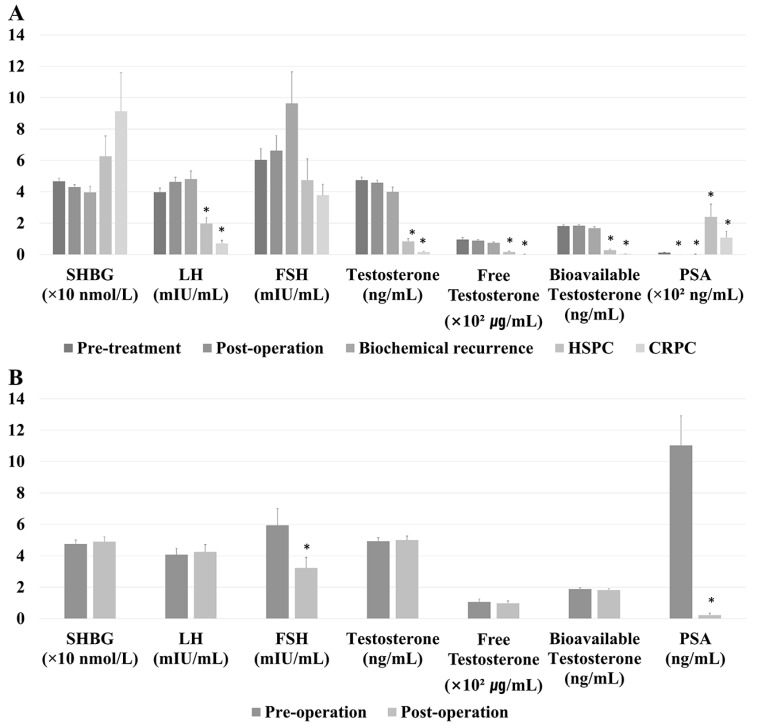
(**A**) Baseline serum hormone concentrations according to tumor state in the androgen deprivation therapy (ADT)-unexposed cohort (n = 225), the hormone-sensitive prostate cancer (HSPC) cohort (n = 59), and the castration-resistant prostate cancer (CRPC) cohort (n = 20). (**B**) Serum hormone concentrations before and after surgery in surgically treated patients (n = 51). * *p* value < 0.05.

Figure 2A displays baseline sex hormone levels according to cancer status in the ADT-unexposed cohort and in patients with HSPC and CRPC (n = 304). Pre-treatment SHBG and FSH levels did not differ significantly. LH levels were significantly lower in patients with HSPC (1.970 ± 2.890 mIU/mL, *p* < 0.001) and CRPC (0.718 ± 0.854 mIU/mL, *p* < 0.001) than in ADT-unexposed patients (3.978 ± 2.206 mIU/mL). Total testosterone, free testosterone, and bioavailable testosterone levels, which were 4.747 ± 1.625 ng/mL, 0.096 ± 0.107 ng/mL, and 1.822 ± 0.554 ng/mL, respectively, in the ADT-unexposed cohort were each significantly lower in the HSPC (0.840 ± 1.415 ng/mL, 0.017 ± 0.036 ng/mL, and 0.280 ± 0.480 ng/mL, respectively) and CRPC (0.170 ± 0.135 ng/mL, 0.019 ± 0.002 ng/mL, and 0.036 ± 0.032 ng/mL, respectively) groups (*p* < 0.001 each). Compared with PSA levels in the pre-treatment cohort (12.709 ± 13.273 ng/mL), PSA levels were significantly lower in the post-operative (0.354 ± 1.335 ng/mL, *p* < 0.001) and BCR (1.823 ± 4.728 ng/mL, *p* < 0.001) groups and significantly higher in the HSPC (240.792 ± 620.381 ng/mL, *p* < 0.001) and CRPC (108.413 ± 163.057 ng/mL, *p* < 0.001) groups. 

Figure 2B shows serum sex hormone concentrations before and after surgery in the same group of patients (n = 51). FSH (3.229 ± 4.486 vs. 5.941 ± 7.044 mIU/mL, *p* < 0.001) and PSA (0.222 ± 0.947 vs. 11.039 ± 13.323 ng/mL, *p* < 0.001) concentrations were significantly lower after compared to before the operation.

**Figure 3:**
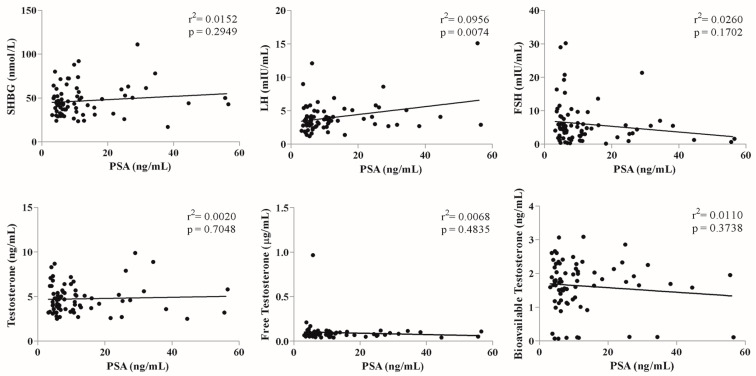
Correlation between sex hormones and PSA levels in pre-treatment cohort (n = 74). * *p* value < 0.05.

Figure 3 displays the correlation between sex hormones and PSA levels in the pre-treatment cohort. There was a significant positive correlation between LH and PSA level (r2 = 0.0956, *p* = 0.0074). However, other hormone levels did not show significant correlations.

**Figure 4:**
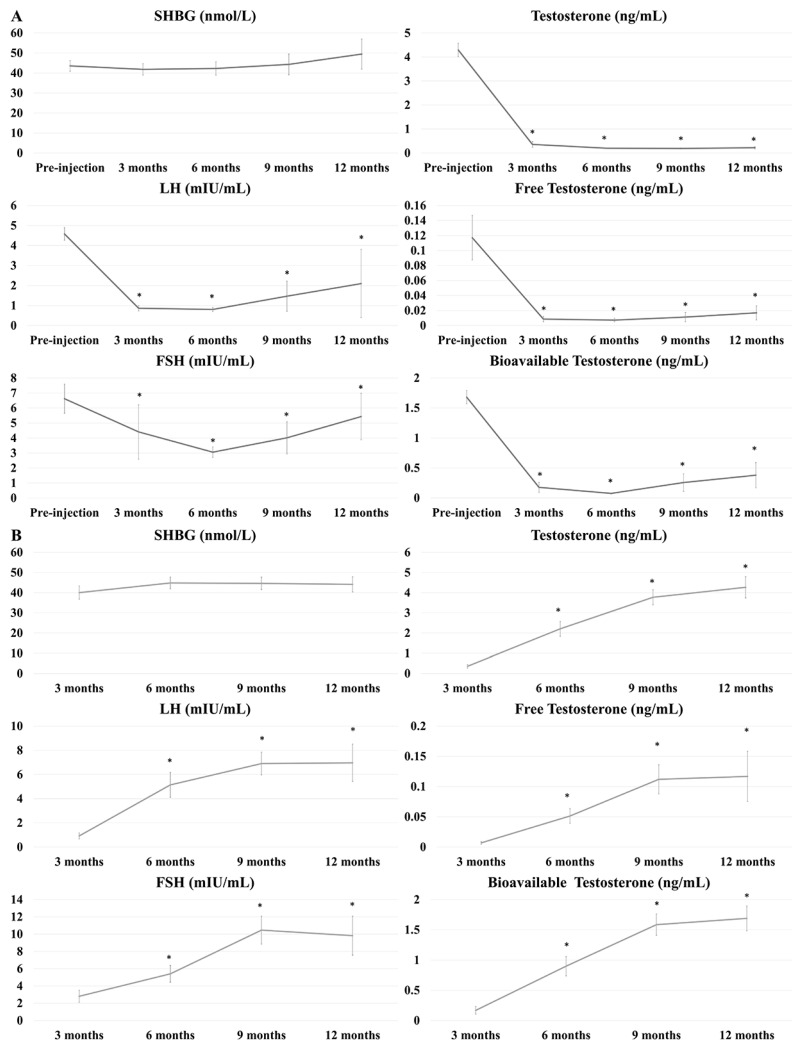
Serum hormone concentrations (**A**) after receiving androgen deprivation therapy (ADT) (n = 57) and (**B**) after the withdrawal of ADT (n = 38). * *p* value < 0.05.

Figure 4A shows changes in serum levels of sex hormones after ADT with LHRH agonist (n = 45) and LHRH antagonist (n = 12). Serum levels of all hormones tested, except SHBG, were significantly decreased after the start of ADT (*p* < 0.01 each). FSH levels differed significantly in patients treated with LHRH agonist and LHRH antagonist after 3 (4.408 ± 11.903 vs. 1.294 ± 2.081, *p* < 0.01), 6 (3.062 ± 2.065 vs. 1.221 ± 1.453, *p* < 0.01), 9 (4.014 ± 4.822 vs. 0.562 ± 0.371, *p* < 0.01), and 12 (5.433 ± 4.661 vs. 0.713 ± 0.807, *p* < 0.01) months. Figure 4B shows serum hormone levels after the withdrawal of ADT (n = 38). Serum concentrations of all hormones tested, except SHBG, were significantly higher 3 months after than at the time of ADT discontinuation (*p* < 0.01 each). 

Pathologic examination after surgery of the pre-treatment group that included the ADT-unexposed cohort (n = 74) showed that 58.1% had pathologic grade ≥3, 44.6% had T3 stage, and 1.4% had N1 stage (Table 2). Fifty patients (67.6%) had unfavorable pathology. Multivariate analysis showed that a high LH level (odds ratio (OR): 1.59, 95% confidence interval (CI): 1.03–2.47, *p* = 0.0376) and high PSA level (OR: 1.13, 95% CI: 1.03–1.24, *p* = 0.0133) were significantly associated with a high risk of unfavorable pathology (Table 3).

## 4. Discussion

In this study, baseline serum hormone levels were assessed in prostate cancer patients at various levels of treatment. LH, total testosterone, free testosterone, and bioavailable testosterone concentrations were lower in the HSPC and CRPC groups, likely because almost all were receiving ADT at the time of sampling. A comparison of pre-operative and post-operative (median 120 days after the operation; post-operation lab was usually conducted at the second visit) serum hormone concentrations in a group of 51 patients showed that FSH and PSA levels were significantly lower after surgery. FSH was reported to be present in the cytoplasm of human prostate tumor tissue and metastatic lymph nodes and to be synthesized even following ADT, suggesting that the prostate is an extra-pituitary source of FSH [11]. In our study, tumor volume significantly correlated with a change of FSH concentration after prostatectomy (rho = −0.640, *p* = 0.014, data not shown), suggesting that the post-operative reduction in FSH may be associated with the removal of tumor tissue. In addition, FSH receptors were detected in higher percentages of prostate cancer tissue than normal prostate tissue and benign prostatic hyperplasia [12]. In our study, the FSH level was higher in patients treated with LHRH agonist than with LHRH antagonist, which is in agreement with previous findings [13]. LHRH antagonists may directly inhibit LHRH receptors in extra-pituitary tissues, such as the testes, and the serum level of inhibin B that inhibited the secretion of FSH production was lower in patients treated with LHRH agonist than LHRH antagonist [14,15]. Repeated administration of LHRH agonist may also induce a microsurge [2]. FSH concentration may be related to tumor volume, suggesting a correlation before treatment. Further studies, however, are needed to understand the differences in FSH concentration between patients treated with LHRH agonists and antagonists. 

We also found that baseline LH levels in the pre-treatment cohort were associated with an unfavorable pathology. LH receptor is present in human prostate epithelial cells, but LH levels are lower in prostate cancer cells than in benign prostatic hyperplasia [16]. LH receptor levels are higher in hormone sensitive than in hormone insensitive cell lines [16]. Higher LH levels in older men may have an effect on the development of benign prostatic hyperplasia or prostate carcinoma [16]. Exposure of cancer cell lines to LH was associated with up-regulation of steroidogenesis within the tumor [17]. Increased steroidogenesis has been frequently observed in CRPC cells, with intratumoral steroidogenesis associated with resistance to ADT [18]. Most studies assessing the role of LH in prostate cancer have been in vitro studies in cancer cell lines, with few clinical studies assessing LH in prostate cancer, except in patients receiving ADT. A comparison of patients with prostate cancer having PSA ≥4 ng/mL with patients having benign prostatic hyperplasia found that serum LH level was lower in the former [19]. Baseline LH level may be associated with Gleason grade or TN stage, which may be helpful as a criterion for active surveillance. In addition, our study showed a positive correlation between LH and PSA levels.

Testosterone is a growth factor for prostate cancer [20]. A meta-analysis showed a complex interplay between serum testosterone level and tumor biology, with serum testosterone not being prognostic for survival or BCR in patients with localized prostate cancer [21]. Low levels of bioavailable and free testosterone have been associated with high grade tumors [22]. Hypogonadism has been frequently observed in patients with other advanced cancers, but its cause may be primary or secondary [23]. In addition, hypogonadism in advanced cancer patients may be associated with low serum albumin resulting from the depletion of body protein [24]. Bioavailable testosterone is composed of free testosterone and albumin-bound testosterone, the latter of which can easily dissociate from albumin and have biological activity. SHBG also binds to testosterone with high affinity. High SHBG level has been associated with extraprostatic extension [25]. However, another study reported that SHBG level was not predictive of the diagnosis or aggressiveness of prostate cancer [26].

This study has several limitations. First, its retrospective design may have introduced selection bias. However, the serum concentrations of all hormones were measured prospectively. Because the total cohort consisted of many stages of disease, it was classified into various sub-cohorts. The small cohort of withdrawing the ADT consisted of patients who underwent neoadjuvant hormonal therapy. A few patients (n = 13) underwent radiotherapy in the total cohort, but we did not analyze it. Second, sex hormone concentrations may be influenced by various confounding factors, such as age, comorbidity, and circadian rhythms, as well as by tumor stage. In addition, retrospective data collection did not guarantee the same timepoint of sample collection. However, clinical data about sex hormones except testosterone were very rare. Systematic prospective study about these hormones will provide an answer regarding the new biomarker and mechanism in prostate cancer.

## 5. Conclusions

The concentrations of sex hormones, including LH, FSH, and testosterones, are affected by ADT in prostate cancer patients. FSH levels decreased after radical prostatectomy. LH levels was positively correlated with PSA levels. High baseline LH prior to treatment of the ADT-unexposed cohort was associated with unfavorable pathology. Further studies are needed to assess the roles of sex hormones in the development of prostate cancer. 

## Figures and Tables

**Table 1:** Baseline characteristics.

	Total Cohort (n = 415)	ADT-Unexposed Cohort (n = 225)
	Mean ± SD or Number (%)	Mean ± SD or Number (%)
Age at diagnosis (years)	67.3 ± 7.6	65.7 ± 6.5
Age at the first exam (years)	68.5 ± 7.4	66.8 ± 6.5
5α-Reductase inhibitor history	32 (7.7%)	18 (8.0%)
PSA level (ng/mL)	73.5 ± 287.7	11.8 ± 16.7
Grade group at biopsy		
- ≤2	157 (37.8%)	124 (55.1%)
- ≥3	224 (54.0%)	99 (44.0%)
Percent of positive core (%)	49.0 ± 34.3	34.1 ± 24.1
Maximum percent of positive core (%)	53.9 ± 32.5	41.1 ± 27.0
Condition at the first exam		
- Pre-treatment	130 (31.3%)	74 (32.8%)
- Post-operation	171 (41.2%)	133 (59.1%)
- Biochemical recurrence	35 (8.4%)	18 (8.0%)
- Hormone-sensitive prostate cancer	59 (14.2%)	0 (0.0%)
- Castration-resistant prostate cancer	20 (4.8%)	0 (0.0%)
Medication at the first exam		
- None	266 (64.1%)	225 (100.0%)
- LHRH agonist	115 (27.7%)	0 (0.0%)
- LHRH antagonist	10 (2.4%)	0 (0.0%)
Clinical T stage		
- 2	227 (54.7%)	158 (70.9%)
- 3a	100 (24.1%)	51 (22.9%)
- 3b	51 (12.3%)	10 (4.5%)
- 4	26 (6.3%)	4 (1.8%)
Clinical N1 stage	68 (16.7%)	1 (0.4%)
Clinical M1 stage	63 (15.5%)	0 (0.0%)
Prostate volume (cc)	35.5 ± 20.6	32.8 ± 17.5

ADT androgen deprivation therapy, SD standard deviation, PSA prostate specific antigen, LHRH luteinizing hormone-releasing hormone.

**Table 2:** Pathologic characteristics in the pre-treatment cohort (n = 74).

	Mean ± SD or Number (%)
Grade group	
- ≤2	31 (41.9%)
- ≥3	43 (58.1%)
Pathologic T stage	
- ≤2	41 (55.5%)
- 3a	22 (29.7%)
- 3b	11 (14.9%)
Pathologic N1 stage	1 (1.4%)
Tumor percent (%)	18.3 ± 17.6
Tumor volume (cc)	5.4 ± 6.4
Perineural invasion	51 (68.9%)
Lymphovascular invasion	23 (31.1%)
Unfavorable pathology	50 (67.6%)

ADT androgen deprivation therapy, SD standard deviation.

**Table 3:** Logistic regression analysis to predict unfavorable pathology in the pre-treatment cohort (n = 74).

	Univariate				Multivariate			
	OR	95%	CI	*p*	OR	95%	CI	*p*
SHBG level	1.01	0.98	1.04	0.4439				
LH level	1.53	1.02	2.29	0.0397	1.59	1.03	2.47	0.0376
FSH level	1.01	0.93	1.09	0.8398				
Testosterone level	1.00	0.74	1.35	0.9957				
Free testosterone level	0.01	0.00	558.61	0.3698				
Bioavailable testosterone level	0.70	0.29	1.69	0.4334				
Preoperative PSA level	1.10	1.02	1.19	0.0124	1.13	1.03	1.24	0.0133

ADT androgen deprivation therapy, SHBG sex hormone-binding globulin, LH luteinizing hormone, FSH follicle-stimulating hormone, PSA prostate specific antigen.

## Abbreviations

ADT	androgen deprivation therapy
BCR	biochemical recurrence
CI	confidence interval
CRPC	castration-resistant prostate cancer
FSH	follicle-stimulating hormone
HSPC	hormone-sensitive prostate cancer
LH	luteinizing hormone
LHRH	luteinizing hormone releasing hormone
OR	odds ratio
PSA	Prostate-specific antigen
SHBG	sex hormone-binding globulin
